# Carcinome micropapillaire invasif: une tumeur mammaire rare et aggressive

**DOI:** 10.11604/pamj.2021.40.29.31348

**Published:** 2021-09-09

**Authors:** Faten Limaïem, Saâdia Bouraoui

**Affiliations:** 1 Université de Tunis El Manar, Faculté de Médecine de Tunis, Tunis, Tunisie

**Keywords:** Sein, cancer, carcinome micropapillaire, Breast, cancer, micropapillary carcinoma

## Abstract

Invasive micropapillary carcinoma of the breast is a rare and distinct histological variant of breast cancers, accounting for less than 3% of all breast carcinomas. It is characterized by a pejorative prognosis due to heavy lymph node involvement and the presence of numerous vascular and lymphatic emboli. We here report the case of a 63-year-old woman presenting with left mastodynia evolving for two months. Physical examination revealed breast asymmetry with skin retraction and multiple suspected lymph nodes in the left axilla. Mammography objectified a spiculated mass in the left breast classified as ACR5. Breast microbiopsy showed invasive micropapillary carcinoma. The patient underwent left Patey’s mastectomy with ipsilateral axillary dissection. Macroscopically, the tumor was grayish-white with spiculated margins located at the union of the two external quadrants and measuring 13 x 8 cm (Panel A). Histological examination of the surgical specimen revealed invasive carcinomatous proliferation arranged in clusters, nests, micropapillae, morules and glands with reversed polarity within a fibro-inflammatory stroma retracted around the carcinomatous structures (Panel B, C). Histoprognostic grading (SBR grade) modified according to ELSTON and ELLIS was 3. Several peritumoral vascular emboli were detected as well as lymph nodes metastases 20N+/20N. Immunohistochemistry using EMA showed reversed polarity (Figure D). Molecular classification of the tumor was luminal B. The postoperative course was simple. The patient underwent adjuvant chemoradiotherapy. Currently, the patient is systematically monitored on an outpatient basis.

## Image en médecine

Le carcinome micropapillaire invasif du sein est une variante histologique rare et distincte des cancers du sein représentant moins de 3% de l´ensemble des carcinomes mammaires. Il se caractérise par un pronostic péjoratif en raison d´un envahissement ganglionnaire massif et de la présence de nombreux emboles vasculaires et lymphatiques. Il s´agit d´une femme âgée de 63 ans ayant consulté pour mastodynie gauche évoluant depuis deux mois. L´examen physique a révélé une asymétrie mammaire avec rétraction cutanée et de multiples adénopathies axillaires gauches suspectes. La mammographie a objectivé une masse spiculée du sein gauche classée ACR5. La microbiopsie mammaire a conclu à un carcinome micropapillaire invasif. La patiente a bénéficié d´une mastectomie gauche de type PATEY avec curage axillaire homolatéral. Macroscopiquement la tumeur était blanc-grisâtre à contours spiculés située à l´union des deux quadrants externes et mesurait 13 x 8 cm (A). L´examen histologique de la pièce opératoire a révélé une prolifération carcinomateuse infiltrante agencée en amas, nids, micro-papilles, morules et glandes à polarité inversée au sein d´un stroma fibro-inflammatoire rétracté au pourtour des structures carcinomateuses (B, C). Le grade histopronostique SBR modifié selon ELSTON et ELLIS était de 3. De nombreux emboles vasculaires péri-tumoraux ont été retrouvés ainsi que des métastases ganglionnaires 20N+/20N. L´étude immunohistochimique moyennant l´EMA a objectivé une inversion de la polarité (D). La classification moléculaire de la tumeur était luminal B. Les suites opératoires étaient simples. La patiente a reçu une radio-chimiothérapie adjuvante. Elle est actuellement régulièrement suivie en consultation externe.

**Figure 1:**
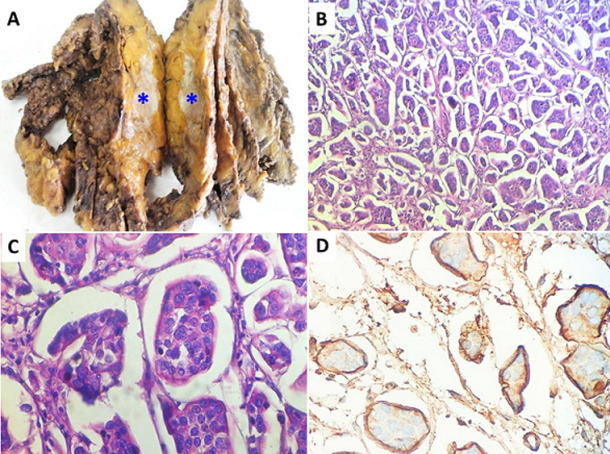
A) examen macroscopique de la pièce de mastectomie révélant une masse tumorale blanc-grisâtre mal limitée mesurant 13 x 8 cm (astérisque bleue); B) prolifération carcinomateuse agencée en morules cernées d´un espace clair optiquement vide (Hématoxyline et éosine, x40); C) Amas et nids de cellules carcinomateuses cernés par un espace lacunaire avec inversion de la polarité (Hématoxyline et éosine, x400); D) positivité membranaire périphérique avec l´anticorps anti-EMA soulignant l´inversion de la polarité (Immunohistochimie, x400)

